# Toll and IMD Immune Pathways Are Important Antifungal Defense Components in a Pupal Parasitoid, *Pteromalus puparum*

**DOI:** 10.3390/ijms241814088

**Published:** 2023-09-14

**Authors:** Lei Yang, Lei Yang, Xiaofu Wang, Cheng Peng, Xiaoyun Chen, Wei Wei, Xiaoli Xu, Gongyin Ye, Junfeng Xu

**Affiliations:** 1 State Key Laboratory for Managing Biotic and Chemical Threats to the Quality and Safety of Agro-Products, Key Laboratory of Traceability for Agricultural Genetically Modified Organisms, Ministry of Agriculture and Rural Affairs, Zhejiang Academy of Agricultural Sciences, Hangzhou 310021, China; 2 State Key Laboratory of Rice Biology and Breeding & Ministry of Agricultural and Rural Affairs Key Laboratory of Molecular Biology of Crop Pathogens and Insects, Institute of Insect Sciences, Zhejiang University, Hangzhou 310058, China

**Keywords:** parasitoid, Toll pathway, IMD pathway, entomopathogenic fungi, scanning electron microscopy (SEM)

## Abstract

Insects employ multifaceted strategies to combat invading fungi, with immunity being a promising mechanism. Immune pathways function in signal transduction and amplification, ultimately leading to the activation of antimicrobial peptides (AMPs). Although several studies have shown that immune pathways are responsible for defending against fungi, the roles of parasitoid immune pathways involved in antifungal responses remain unknown. In this study, we evaluated the roles of the Toll and IMD pathways of a pupal parasitoid, *Pteromalus puparum* (Hymenoptera: Pteromalidae), in fighting against *Beauveria bassiana* (Hypocreales: Cordycipitaceae). Successful colonization of *B. bassiana* on *P. puparum* adults was confirmed by scanning electron microscopy (SEM). AMPs were induced upon *B. bassiana* infection. The knockdown of key genes, *PpTollA* and *PpIMD*, in Toll and IMD signaling pathways, respectively, significantly compromised insect defense against fungal infection. The knockdown of either *PpTollA* or *PpIMD* in *P. puparum* dramatically promoted the proliferation of *B. bassiana*, resulting in a decreased survival rate and downregulated expression levels of AMPs against *B. bassiana* compared to controls. These data indicated that PpTollA and PpIMD participate in Toll and IMD-mediated activation of antifungal responses, respectively. In summary, this study has greatly broadened our knowledge of the parasitoid antifungal immunity against fungi.

## 1. Introduction

Entomopathogenic fungi are viewed as effective substitutes for chemical insecticides due to their broad host range, environmental friendliness, and higher safety to non-target organisms [1]. One of the most widely used fungi for biocontrol of pest insects is *Beauveria bassiana* (Hypocreales: Cordycipitaceae). This fungus parasitizes over 700 insect species in 149 families and has been registered worldwide as a mycoinsecticide formulation [2]. Direct invasion of the epidermis is the main way that entomopathogenic fungi infect insects, with *B. bassiana* conidia adhering to the insect’s epicuticle, developing germ tubes, and colonizing the bodies of their hosts by breaking through the cuticle with the aid of degrading enzymes [3,4].

Insects have evolved various strategies to fight against fungal infection, among these the innate immune system playing a significant role in antifungal responses [5]. Once fungal conidia penetrate the host cuticular layers, the cellular and humoral immunity of the host are stimulated. Hemocytes mediate cellular immune responses, inducing phagocytosis, encapsulation, and coagulation, while humoral immunity relies on multiple signal transduction pathways, inducing the expression levels of antimicrobial peptides (AMPs) and the activation of the phenoloxidase cascade [6,7].

The Toll and IMD pathways are two core signaling pathways involved in the antifungal immune response [8,9]. Typically, the Toll pathway is a major immune pathway against fungi and Gram-positive bacteria, with the recognition molecules, such as S-PGRP, βGRP, and GNBP, participating in this process. They adhere to the pathogen-associated molecular patterns of microbes, leading to the cleavage of the inactive form of Späzle (Spz) and the recruitment of the Toll receptor. An intracellular signal cascade transmitted through the Toll receptor eventually causes the release of Dorsal and Dif [10,11]. In contrast, the IMD pathway responds to Gram-negative bacteria and is initiated by PGRP-L type genes, which activate IMD, FADD, and Dredd sequentially and further phosphorylate Relish. The release of NF-κB transcription factors (Dorsal, Dif, and Relish) induce the nuclear translocation [12,13].

Transcriptome analysis has demonstrated that regulators in immune pathways can be activated upon *B. bassiana* challenge and the Toll signaling pathway was strongly activated during fungal penetration stage [14,15]. Moreover, *Spz* or dorsal-related immunity factor (*Dif*) mutant files became more sensitive to fungal infection than wild-type files [16]. *REL1*, a dorsal homologous gene, has been identified in two mosquitos and their roles in anti-*B. bassiana* have been affirmed [17,18]. RNA silencing of *AgREL1* and *AaREL1* revealed their functions in inducing the expression of antifungal peptides and increasing susceptibility to *B. bassiana*. Previous studies have also suggested the involvement of the IMD pathway in the antifungal response [15,19]. For instance, *Drosophila* IMD pathway-derived AMPs were stimulated by fungal infection [19]. *Relish* knockdown flies were more sensitive to natural fungal infection [20]. Silencing of the REL2 genes impaired the lifespan of mosquitos, whereas a slight but not significant increase in survival has been shown in *caspar*-mutant mosquitos since caspar could block the proteolytic activation of Relish [21,22].

Parasitoids are significant natural controls of their insect hosts and are considered as important agents in integrated pest management (IPM) programs. Due to the widespread pathogenicity of *B. bassiana*, it may affect the survival rates of the parasitoid wasps [23,24]. The combined use of microbes and parasitoids may enhance the effectiveness of IPM programs. However, it is essential to fully understand the complex interactions between parasitoids and entomopathogenic fungi. The immune system is a potent defense of parasitoid wasps in repelling the invading fungi. There are limited studies showing the expression profiles of parasitoid immune signaling molecules upon fungal challenge, let alone the immune mechanisms of parasitoids against fungi. *Pteromalus puparum* (Hymenoptera: Pteromalidae) is a pupal endoparasitoid wasp that can parasitize numerous hosts, including more than 20 species [25]. It can serve as a promising biological tool in agriculture. A high-quality chromosome-level *P. puparum* genome was published previously, along with the identification of immune genes [26,27], laying a foundation for elucidating its antifungal mechanism.

In this work, we silenced the key regulators (*PpTollA* and *PpIMD*) in the Toll and IMD pathways to investigate their roles in antifungal response. The results showed that either knockdown *PpTollA* or *PpIMD* dramatically promoted the proliferation of *B. bassiana* and decreased the expression levels of AMPs following fungal challenge, leading to an increase in susceptibility to *B. bassiana*.

## 2. Results

### 2.1. The Successful Invasion of B. bassiana Activated Host Antifungal Immune Responses

**Figure 1:**
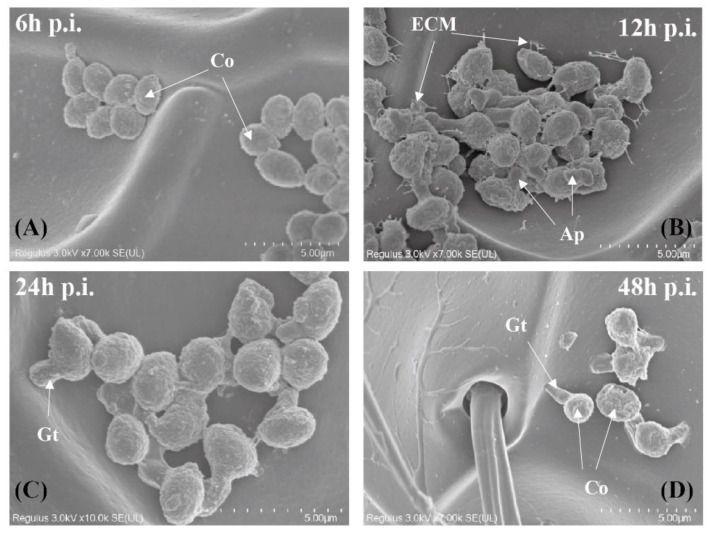
Observation of the attachment and germination of *B. bassiana* conidia on the cuticle surface of *P. puparum* adults. *P. puparum* wasps were inoculated with *B. bassiana* ARSEF 2860 by spraying a conidial suspension. (**A**) Conidia (Co) attached to the cuticle of host and some gemmiform protuberances appeared on some conidia after 6 h of inoculation. (**B**) Germinated conidia secreted extracellular mucilage (ECM) and formed appressoria (Ap) after 12 h of inoculation. (**C**) Conidia germinated unidirectionally or bidirectionally to form germ tubes (Gt). (**D**) The germ tube pinned directly into the cuticle of the host.

The adhesion of *B. bassiana* conidia onto *P. puparum* cuticle was observed using a scanning electron microscope (SEM) (Figure 1). After 6 h incubation, most attached spores retained smooth, round, and plump shapes with the appearance of gemmiform protuberances on some areas of the spores (Figure 1A). As time progressed, the morphology of *B. bassiana* spores exhibited evident changes. After 12 h, the spores became rough and uneven due to the secretion of extracellular mucilage and the production of appressoria (Figure 1B). Sustained appressorial growth led to the formation of germ tubes 24 h post infection (p.i.), eventually leading to the penetration of the parasitoid’s cuticle (48 h p.i.) (Figure 1C,D). These observations shed light on the initial development processes of conidia through spraying.

**Figure 2:**
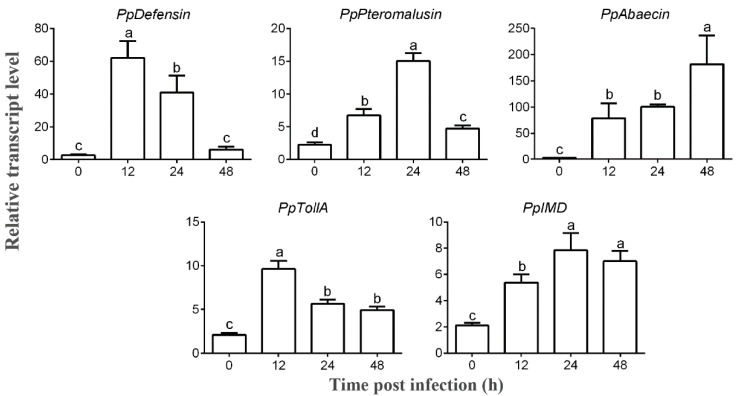
The elicitation of antimicrobial peptide (AMP) genes and key genes in immune pathways followed *B. bassiana* infection. The expression levels of these parasitoid genes were detected at 0, 12, 24, and 48 h p.i. Error bars represent the means ± standard deviations from three biological replicates. Statistical analysis is conducted by one-way analysis of variance (ANOVA) followed by Tukey’s test. The significant difference is showed by different lowercase letter (a–d) (*p* < 0.05).

**Figure 3:**
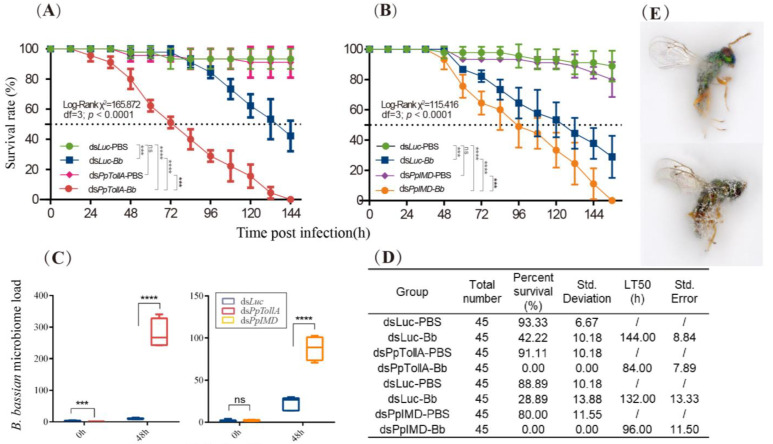
Survival curves for *P. puparum* adults treated with ds*PpTollA* (**A**) or ds*PpIMD* (**B**), ds*Luc* with or without *B. bassiana* inoculation. Log-rank test was used to assess differences in survival rates between different treatments. Significant differences were represented by definite *p* value and asterisk (*** *p* < 0.001; **** *p* < 0.0001; ns, not significant, *p* > 0.05). (**C**) Fungal load estimate in *P. puparum* adults at 0 h and 48 h p.i. via relative quantification of fungal *18s* rRNA. Data were analyzed by Student *t* test (*** *p* < 0.001; **** *p* < 0.0001, ns, not significant, *p* > 0.05). Error bars represent the means ± standard deviations from three biological replicates. (**D**) *P. puparum* median lethal time (LT50) and survival rate under RNA knockdown and *B. bassiana* infection. (**E**) The view of dead *P. puparum* symptoms induced by fungal infection. ds*Luc* (ds*PpTollA*/ds*PpIMD*)-PBS, *P. puparum* adults treated with ds*Luc* (ds*PpTollA*/ds*PpIMD*) followed by PBS spraying. ds*Luc* (ds*PpTollA*/ds*PpIMD*)-*Bb*, *P. puparum* adults treated with ds*Luc* (ds*PpTollA*/ds*PpIMD*) followed by *B. bassiana* infection.

Furthermore, we evaluated the elicitation of AMPs and key signaling molecules (*PpTollA* and *PpIMD*) of immune pathways upon *B. bassiana* infection in *P. puparum* adults. The expression levels of *P. puparum* AMPs were dramatically increased, reaching a peak at different time points after infection followed by a significant decrease as shown in Figure 2, which indicated the activation of immune system. Similarly, the expression levels of *PpTollA* and *PpIMD* were elevated after infection. Dead adult wasps affected by *B. bassiana* were transferred to the Petri dishes containing moist filter paper to check fungal growth. Figure 3E depicts the morphology of *P. puparum* after death. The growth and sporulation of the hyphae out of the cuticle confirmed that fungal infection was the cause of death.

### 2.2. The Roles of PpTollA on B. bassiana Infection

The inoculation dosage of fungi can greatly affect its pathogenicity towards parasitoids. Therefore, it is essential to determine the optimal dosage that guarantees efficient invasion while avoiding excessive or insufficient mortality of the parasitoids in subsequent experiments. With this in mind, we inoculated ds*Luc*-silenced *P. puparum* with varied concentrations of *B. bassiana* (Figure S1). The survival rate of adult wasps demonstrated a dose-dependent decrease, indicating that higher spraying concentrations of *B. bassiana* lead to higher death rates of *P. puparum*. Based on these observations, we determined that a dosage of either 10 or 20 conidia/mm^2^ of *B. bassiana* was suitable for ensuring recording time and effective invasion. We selected a spraying concentration of 20 conidia/mm^2^ for the subsequent experiments.

**Figure 4:**
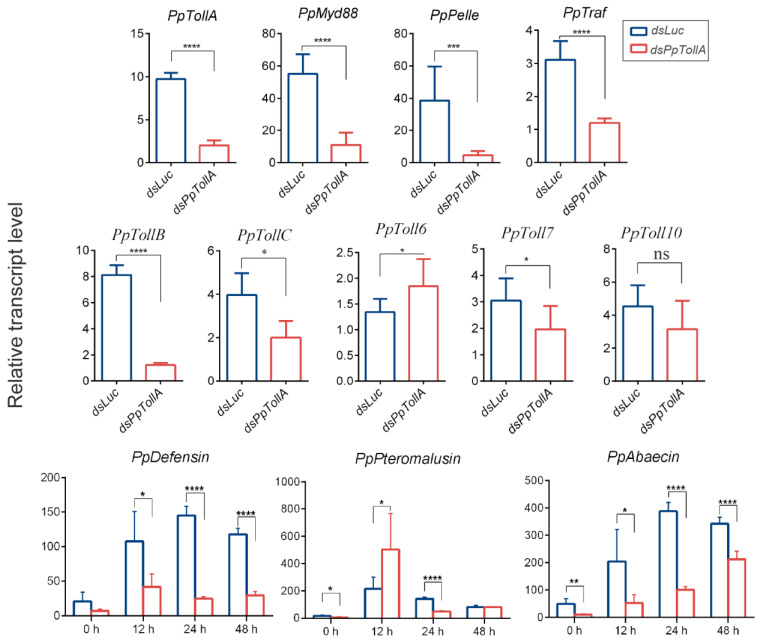
The expression profiles of genes in ds*PpTollA*-silenced and ds*Luc*-silenced *P. puparum* by qRT-PCR. Significant differences were represented by definite *p* value and asterisk (* *p* < 0.05; ** *p* < 0.01; *** *p* < 0.001; **** *p* < 0.0001; ns, not significant, *p* > 0.05). Error bars represent the means ± standard deviations from three biological replicates. The *18s* rRNA gene was used as internal control.

We selected the *PpTollA* as a critical component to assess the roles of the Toll pathway in defending against fungal infection. Knockdown experiments were conducted on pupal parasitoids, which were subsequently inoculated with *B. bassiana* upon emergence. Microinjections of ds*PpTollA* resulted in an 80% reduction in *PpTollA* gene expression compared to that in ds*Luc*-treated parasitoids (Figure 4). The relative expression levels of the other five PpToll-like genes were also evaluated. Significantly downregulated expression levels were observed in *PpTollB*, *PpTollC*, and *PpToll7* (Figure 4). Combined with their basal expression levels in the adult stage of *P. puparum* (Table S1), the successful silencing of *PpTollA* substantially suppressed the overall expression of Toll genes in *P. puparum* and consequently had a severe impact on Toll pathway. Moreover, downstream components, such as *PpMyd88*, *PpPelle*, and *PpTraf*, also exhibited suppressed expression levels (Figure 4). The survival of insects (ds*PpTollA*-*Bb*) significantly differed from that of other treatments (Figure 3A). The LT50 of ds*PpTollA-Bb* group was 84.00 h, which is significantly lower than that of the ds*PpTollA*-PBS group (144.00 h) (Figure 3D).

To confirm whether the reduced survival is correlated with fungal proliferation in *PpTollA* knockdown parasitoids, we assessed fungal load 48 h after inoculation (Figure 3C). It showed a significantly higher number of fungal genome copies in ds*PpTollA-Bb*-treated insects compared to ds*Luc-Bb*-infected insects. Additionally, we investigated the abundance of AMP genes at different time points post fungal infection (Figure 4). *PpDefensin* and *PpAbaecin* were significantly downregulated in *PpTollA*-knockdown parasitoids, while the expression profile of *PpPteromalusin* appeared unaffected.

### 2.3. The Roles of PpIMD on B. bassiana Infection

**Figure 5:**
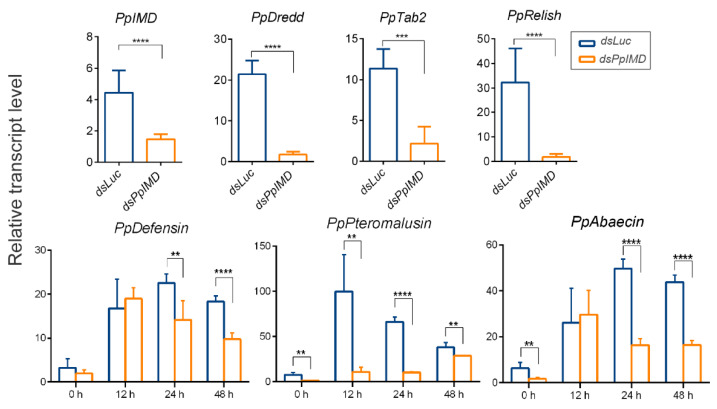
The expression profiles of genes in ds*IMD-*silenced and ds*Luc*-silenced *P. puparum* by qRT-PCR. Significant differences were represented by definite *p* value and asterisk (** *p* < 0.01; *** *p* < 0.001; **** *p* < 0.0001). Error bars represent the means ± standard deviations from three biological replicates. The *18s* rRNA gene was used as internal control.

The susceptibility of *PpIMD*-silenced parasitoids was monitored after the fungal challenge. A significant reduction was observed in the transcription level of *PpIMD* in *PpIMD* dsRNA-treated wasps (with a 67% silencing efficiency) compared to the control groups (Figure 5). Furthermore, we detected remarkable decreases in the expression levels of *PpDredd*, *PpTab2*, and *PpRelish* following treatment with *PpIMD* dsRNA (Figure 5). The *PpIMD* knockdown wasps displayed higher sensitivity to fungal infection in comparison to ds*Luc*-treated parasitoids. The LT50 for the former group (96.00 h) was significantly shorter than that of the latter group after the *B. bassiana* infection (LT50 = 132.00 h), indicating that PpIMD plays an essential role in antifungal defense against pathogens in parasitoids (Figure 3D). Additionally, the transcription of *PpPteromalusin* was significantly suppressed, and the suppression effect was stronger than that of *PpTollA*-interfered parasitoid wasps. In contrast, the expression levels of *PpDefensin* and *PpAbaecin* were significantly suppressed 24 h p.i. (Figure 5). The significant decreases in AMPs coincided with a significant increase in fungal load (Figure 3C), which indicated that silencing of *PpIMD* resulted in several-folds increase in fungal load 48 h post *B. bassiana* infection.

## 3. Discussion

In this work, we confirmed the successful colonization of *B. bassiana* into *P. puparum* adults and eventually leading to the death of the host. Our analysis revealed that germinated conidia form appressoria within 12 h and germ tube pinned into the cuticle of *P. puparum* within 48 h (Figure 1). We observed that the host immune response was induced at 12 h post infection (Figure 2), indicating that the parasitoid’s immune system was activated once conidia developed. Above all, we evaluate the roles of both Toll and IMD pathways in *P. puparum* by *PpTollA* and *PpIMD* knockdown. Silencing the core genes of these immune pathways, either *PpTollA* or *PpIMD*, significantly inhibited the expression of downstream effectors and compromised host defenses against fungal infections along with a reduced survival (Figure 3, Figure 4 and Figure 5). Collectively, our research provides critical insights into the mechanisms of antifungal immunity in parasitoids and contributes to a comprehensive understanding of insect-fungal molecular interactions.

The successful evasion and completion of a fungal pathogen’s life cycle on a host is highly dependent on its ability to parasitize the host epidermis [28]. In order to assess the infection process of entomopathogenic fungi, SEM has been widely employed. The infection processes of *B. bassiana* were documented via SEM on several insects, including *Rhynchophorus ferrugineus* (Coleoptera: Curculionidae), *Ostrinia nubilalis* (Lepidoptera: Crambidae), and *Leptinotarsa decemlineata* (Coleoptera: Chrysomelidae) [29,30,31]. These experiments revealed that fungal hyphae grew and penetrated into the cuticles of host insects within 48 h post infection, in alignment with the findings in this study. Given that high humidity is a vital factor for fungal germination [32], all these experiments were conducted under the condition of high humidity to facilitate fungal germination. Further investigation could perform a time-course analysis of the early infection process of *B. bassiana* on *P. puparum* under varied humidity levels, thereby gaining a deeper understanding of *B. bassiana*’s virulence towards host insects in diverse environmental conditions.

The Toll pathway has been considered as the primary defense mechanism against fungal invasion in insects, and much research has been conducted on the antifungal effects of immune signaling molecules involved in this pathway [33,34]. Valuable tools, such as RNA interference or loss-of-function mutants, have been employed to investigate the functions of target genes. For instance, during fungal infection of adult flies, *Drosophila* Toll-1 acted as the elicitor of the antifungal peptide drosomycin [35]. Mutation of the Toll ligand *Spz* significantly abolished *drosomycin* expression and reduced the host resistance to fungal challenge [16]. The crucial roles of Toll and *Spz* in regulating the mosquitoes’ survival and sensitivity to fungal challenge have also been demonstrated, where RNA knockdown of either *Toll5A* or *Spz1C* leads to a decreased expression of *Spn27A* following fungal challenge, thereby increasing its susceptibility to *B. bassiana* [36]. The importance of Rel/NF-κB molecules (*Dif*, *dorsal,* and their homologs) in fighting against *B. bassiana* have been revealed through gene knockdown in various insects, such as *Anopheles gambiae* (Diptera: Culicidae), *Aedes aegypti* (Diptera: Culicidae), *Drosophila melanogaster* (Diptera: Drosophilidae), and *Spodoptera litura* (Lepidoptera: Noctuidae) [16,17,18,37]. Additionally, the roles of other Toll pathway molecules, including *Myd88*, *TRAF*, Clip-serine proteases and their homologs, and *TEP*s, have also been uncovered [35,38,39,40,41]. In our assay, injection of dsRNA effectively suppressed the expression of *PpTollA*, leading to a dramatic reduction in the expression levels of downstream components, including *PpMyD88*, *PpPelle*, and *PpTraf*, along with a reduced survival rate in *P. puparum* adults. Our result coordinated with previous studies and highlighted the importance of parasitoid Toll pathway in defending against *B. bassiana*.

In addition, we investigated the role of the IMD pathway in resisting against *B. bassiana* by *PpIMD* knockdown. The IMD pathway is a well-known defense mechanism against Gram-positive bacteria. Interestingly, Wu et al. [42] demonstrated distinct expression patterns of genes implicated into the Toll and IMD pathways upon the injection of recombinant *Bombyx mori* (Lepidoptera: Bombycidae) apolipophorin-III, which exhibited potent antifungal effects on *B. bassiana*. This injection included the upregulation of *BmMyd88* in the Toll pathway and the downregulation of *BmTak1* in the IMD pathway, highlighting the significance of both the Toll and IMD pathway in fungal defense [42]. However, the systemic expression of *Drosophila* drosomycin was regulated by the Toll pathway activation in fat body and controlled by the IMD pathway in the respiratory tract [43]. Additionally, studies in *B. mori* have shown that different immune pathways mediated the expression of different antifungal peptides, with the IMD pathway specifically controlling the expression of *Bmenbocin 1*, *Bmgloverin 2*, and *Bmattacin 1* [15]. Furthermore, the IMD pathway exhibited an earlier activation than the Toll pathway in response to fungal infections in recent studies, indicating the complexity of antifungal immune responses in insects [44]. In our study, the dramatically decreased expression level of *PpIMD* not only hindered the expression of downstream components, such as *PpDredd*, *PpTab2*, and *PpRelish*, but also significantly compromised the host’s defense against *B. bassiana* infection. These findings emphasize the vital role of the *PpIMD*-mediated IMD pathway in mounting an effective antifungal response.

AMPs are vital downstream products of both Toll and IMD immune pathways, directly functioning in killing microorganisms. In this study, the roles of three types of AMPs, defensin, pteromalusin, and abaecin, were investigated upon *B. bassiana* infection. Previous research indicated that defensin and abaecin were regulated by both Toll and IMD signaling pathways [45]. In contrast, pteromalusin, homologous to nasonin, belongs to a new defensin-like subfamily, and whether the regulatory circuit is controlled by Toll or IMD pathways still remains unknown [46]. Our qRT-PCR results demonstrated a significant increase in AMP expressions post-fungal infection. Additionally, *PpDefensin* and *PpAbaecin* maintained relative low transcription levels in parasitoid adults treated with either ds*PpTollA* or ds*PpIMD* in comparison to those treated with ds*Luc*, even under uninfected conditions. Similar observations were found in *B. mori*, where the disturbed Toll and IMD immune pathways using specific inhibitors resulted in reduced expression of antifungal peptides [15]. Conversely, fruit flies with the mutation in *Caspar*^p1^ displayed significantly higher levels of AMP expressions under both uninfected and infected statuses [47]. In short, these previous studies strongly supported our results that PpTollA and PpIMD play crucial roles in activating immune pathways; the knockdown of these genes indeed inhibited the expression of functional AMPs in both uninfected and infected wasps.

## 4. Materials and Methods

### 4.1. Insect Rearing and Fungi Cultivation

Laboratory colonies of *P. puparum* were initially collected from a cabbage field in the experimental farmland of Zhejiang University in 2012 and were reared on their natural host pupae, *Pieris rapae* (Lepidoptera: Pieridae), under a 14L:10D photoperiod at 25 ± 1 °C and 80% relative humidity [48]. Newly emerged *P. puparum* wasps were reared in finger-type plastic tubes with 20% honey water (*v*:*v*). Two-day-old mated female wasps were used for parasitism. 

The entomopathogenic strain, *B. bassiana* ARSEF Bb2860, was used in the following experiments [49]. Fungi were maintained on Sabouraud dextrose agar (SDAY) at 25 °C for 7 days. The mycelia were removed, and harvested spores were filtrated and dried for further use.

### 4.2. Immune Challenge

We inoculated *P. puparum* adult wasps with *B. bassiana* via spraying exposure assay. Before conducting the experiments, we assayed the spore germination rate of each batch of *B. bassiana* conidia. The viability of *B. bassiana* is measured by counts of germinated conidia in a 24 h liquid culture containing 2% sucrose and 0.5% peptone. Conidia with a germination rate exceeding 90% within 24 h of incubation were used for further experiments [50,51]. The concentration of conidia was determined using a Neubauer counting chamber (Shanghaiqiujing, Shanghai, China) and adjusted to an initial concentration of 1 × 10^8^ conidia/mL before dilution.

To perform the experiment, the wasps were anesthetized for five minutes and carefully placed in sterile 90 mm Petri dishes. Three glass cover slips (20 × 20 mm) were also included in the Petri dish alongside the wasps. Each Petri dish with parasitoids was then subjected to either a spray of the spore suspension or a control solution (0.05% Tween-80) using a handheld Micro Ulva fogger. The spraying solution consisted of 2 mL with a concentration of 1 × 10^7^ conidia/mL. Subsequently, the parasitoids were transferred to finger-type plastic tubes with 20% honey water (*v:v*). The concentration of deposited conidia was checked by counting the number of conidia/mm^2^ using microscopic counts from three glass cover slips placed alongside the wasps during spraying [52,53]. To analyze the expression patterns of AMPs and key signaling molecules of the immune pathway upon *B. bassiana* infection, we collected fifteen adult wasps at each time point (0, 12, 24, and 48 h post challenge).

### 4.3. SEM Observation

A total of 12 infected *P. puparum* wasps were used for SEM analysis. These parasitoids were fixed in 2.5% glutaraldehyde for 24 h at 4 °C, post-fixed in 1% buffer (0.1 M sodium cacodylate). Dehydration of the samples was carried out at 4 °C in acetone grades, starting from 30%, 50%, 70%, 80%, 90%, 95%, and 100%, with each grade lasting for 15 min. The dehydrated samples were then dried with tetra methyl silane for 5–10 min for two times, mounted on a stub, and then coated with gold-palladium using Q150T ES (Quorum, East Sussex, UK) before examination by SEM with the Hitachi Regulus 8100 (Hitachi, Tokyo, Japan).

### 4.4. Preparation of dsRNA

To investigate the effects of *PpTollA* and *PpIMD* on *P. puparum* wasps against *B. bassiana*, RNAi-mediated knockdown of *PpTollA* and *PpIMD* was performed along with the observation of their impact on the survival rates of these parasitoids. The complete coding sequences of *PpTollA* and *PpIMD* were obtained from our previous study [27]. Primers with a T7 promoter sequence were designed to amplify 400–500 bp products from the genome of *P. puparum*. The amplified products were then cloned into the pBM23 Toposmart vector (Biomed, China). The accuracy of the amplified sequences was verified by DNA sequencing. Single stranded RNAs were transcribed using MEGAscript T7 Transcription Kit (Ambion, Austin, TX, USA) and then mixed and annealed to get double stranded RNA following the manufacturer’s protocol. After purification, dsRNAs were verified, quantified, and stored at −80 °C.

### 4.5. RNA Interference

Fifty nanoliters of *PpTollA* or *PpIMD* dsRNA were injected into each yellow pupal stage of *P. puparum* using Nanoinject III (Drummond, Birmingham, AL, USA). The ds*Luc*-injected parasitoid groups served as the controls. The RNA silencing efficiency was evaluated by examining the gene expression levels of randomly selected *P. puparum* wasps after emergence. We collected 5 parasitoid wasps in each replicate, and at least three biological replicates were conducted. Furthermore, these adult wasps were divided into two groups, one group challenged with *B. bassiana* and the other group challenged with sterile PBS. The *B. bassiana* suspension used for formal RNAi experiments consisted of a 2 mL-conidial suspension with a concentration of 1 × 10^7^ conidia/mL. Dead wasps were recorded every 12 h post fungal challenge, and the Kaplan–Meier method was used to analyze the difference of survival rates among these RNA silencing parasitoids [54]. For the survival rates, fifteen adult wasps were included in each replicate, and the experiment was replicated three times. Moreover, to determine the gene transcription levels of the challenged parasitoids, adult wasps were sampled at 0, 12, 24, and 48 h post-challenge. In each replicate, we collected five wasps, and we conducted at least three replicates at each time point.

### 4.6. RNA Extraction and qPCR Analysis

All samples were homogenized in Trizol reagent following the manufacturer’s protocol. The extracted RNA was reversely transcribed into cDNA using PrimeScript™ One Step RT-PCR Kit (Takara, Kyoto, Japan). qRT-PCR was performed to examine the gene expression levels in the collected samples, with *18s rRNA* being used as the internal control. Fungal load abundance of RNAi groups was evaluated by analyzing the transcript abundance of *B. bassiana 18s rRNA* as a fungal-specific primer [55]. All these primers used in the present study were listed in Table S2. qPCR was performed using BIO-RAD Real-Time System (Bio-Rad, Hercules, CA, USA) with the following process: initial denaturation at 95 °C for 30 s, then 40 cycles of denaturation at 95 °C for 5 s, annealing, and extension at 60 °C for 34 s. The melting curves for each PCR reaction were checked to verify the formation of primer dimers or sample contamination.

The expression levels of these genes were quantified using the 2^ΔΔCt^ method. These data were presented as the mean ± standard deviation (SD) with at least three replicates. One-way ANOVA analysis and Tukey’s test were used to compare the differential gene expression levels among different time points. The student *t* test was used to compare the mean of two groups.

## 5. Conclusions

Overall, our study offers a valuable understanding into the mechanisms of antifungal immunity in parasitoids through the Toll and IMD pathways. Both key genes, *PpTollA* and *PpIMD*, in these immune pathways are responsible for host defense against fungal infection. Silencing either of them significantly inhibited the expression of downstream effectors, compromised host defenses against fungal infections, and reduced the survival rates of parasitoids. In addition, our findings shed light on the regulation of AMP expressions in parasitoid antifungal immune responses. These results will contribute to a comprehensive understanding of insect-fungal molecular interactions and facilitate the development of more effective strategies for utilizing both parasitoids and entomopathogenic fungi in controlling fungal pathogens.

## Data Availability

Data are contained within the article.

## Supplementary Materials

The supporting information can be downloaded at: https://www.mdpi.com/article/10.3390/ijms241814088/s1.
