# Supplementary material for: Toll and IMD Immune Pathways Are Important Antifungal Defense Components in a Pupal Parasitoid, Pteromalus puparum
Source: Int J Mol Sci. 2023 Sep 14;24(18):14088. doi: 10.3390/ijms241814088 (PMC10531655; doi:10.3390/ijms241814088)
Supplement: Supplementary file 1 [file ijms-24-14088-s001.zip › ijms-2571742-supplementary.pdf]

Supplemental Tables and figures

(A)

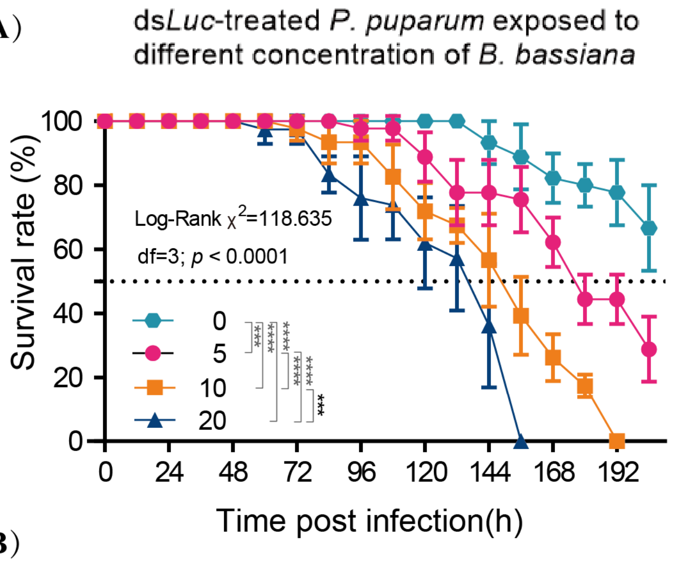

(B)

CONC (Conidia/mm^2)	Total number	Percent survival (%)	Std. Deviation	LT50 (h)	Std. Error
0	45	66.67	13.33	/	/
5	45	28.89	10.18	180.00	5.00
10	46	0.00	0.00	156.00	4.97
20	43	0.00	0.00	144.00	4.23

Figure S1. Virulence of *B. bassiana* to ds*Luc* injected *P. puparum.* The number of dead parasitoids was counted every 12 hours. (A) Survival rate of ds*Luc* treated *P. puparum* in response to different dose (condia/mm^2) of *B. bassiana* at different time points. Log-rank test was used to assess different in survival between treatments. Significant differences were represented by definite *p* value and asterisk (*** $p < 0.001$; **** $p < 0.0001$). (B) Median lethal time (LT50) and percent survival rate of fungal-treated parasitoids.

Table S1. The FPKM values of Toll-like genes in the adult stage of *P. puparum*.

	**gene_id**	**PpAd_1**	**PpAd_2**	**PpAd_3**	**PpAd_4**
PpTollA	PPU12940-RA	49.41966	47.3735	42.18424	59.04608
PpTollB	PPU09907-RA	46.43197	47.72764	36.52581	48.866
PpTollC	PPU09921-RA	5.623631	5.151635	6.790215	5.26776
PpToll6	PPU03159-RA	2.601372	2.317239	5.017081	1.559985
PpToll7	PPU03999-RA	14.01884	13.39101	11.87163	11.97818
PpToll10	PPU00761-RA	0.135699	0	0.135703	0

Table S2. Primers used for qPCR and dsRNA synthesis

**Gene Name**	**Gene ID**		**Sequence (5'-3')**	**Size(bp)**
PpTollA	PPU12940-RA	-F-	CGACATGAACCACAATCGAC	131
		-R-	TCGCCATCGATATACGTCAA	
PpTollB	PPU09907-RA	-F-	CGCGAGATCTTTTGAGAGGC	135

		-R-	TGAGGTTGTTGTTGCTGAGC	
PpTollC	PPU09921-RA	-F-	AACCTCAAGACGCTGCTACT	166
		-R-	AGTTGGACTGGACGAAGGAG	
PpToll6	PPU03159-RA	-F-	CAGCCAGATCGTCTCCATCC	146
		-R-	GTTCTGGCAGGTAGAGCTCC	
PpToll7	PPU03999-RA	-F-	CTGATCGTCCTCGACCTCTC	112
		-R-	TGTGGCTGATCGAGTTGTTC	
PpToll10	PPU00761-RA	-F-	GTCTTCCTGGCCAACGAGT	177
		-R-	CAGACGATGACGGTGCAG	
PpIMD	PPU01186-RA	-F-	AAAGGTGGCACTGGTGAAGT	126
		-R-	TTCGACGCATTCATATTCCA	
PpMyD88	PPU08578-RA	-F-	AGGTTTCAGGACCTCCGAGT	149
		-R-	TTTTCCTGATGCTGTTGCAC	
PpPelle	PPU10296-RA	-F-	GCTGCAACAGACAACTGGAA	127
		-R-	CACAATCAGAACCCCTTGGT	
PpTraf	PPU05667-RA	-F-	TTCTCCCTGGACAAGCACAT	122
		-R-	AGCGACATCTTGTTCATGGC	
PpDredd	PPU06095-RA	-F-	AGCCATGGGTCAGAAGGTG	177
		-R-	TCCTGTGCTTTGTTCTGGAC	
PpTab2	PPU15978-RA	-F-	CGTCTATCAGTGCATTGCCC	168
		-R-	ATCTCGGCGAAATTCAACGG	
PpRelish	PPU06179-RA	-F-	AAAGACTTCGCCGACTACGA	147
		-R-	GATCCGTGATTCGTGTGTTG	
PpPteromalusin	PPU05423-RA	-F-	ACTTCGTTTTCCTGCTCGTC	101
		-R-	TCCCTTCATGATGCAGTCGA	
PpDefensin	PPU12673-RA	-F-	CAGCGCAGAGTAACCTGTGA	168
		-R-	TTATCCAAAGCGCTTGTTCC	
PpAbaecin	PPU09187-RA	-F-	TTGCTCGCTGTAGCCCTTAT	123
		-R-	TCTGCCACTCCAAGTTCCTT	
Pp18s rRNA		-F-	CGAGCGATGAACCGACAG	
		-R-	CGGGGAGGTAGTGACGAA	
Bb18s rRNA		-F-	AGATACCGTCGTAGTCTTAACCATAAACT	
		-R-	TTCAGCCTTGCGACCATACT	
dsPpTollA	PPU12940-RA	-F-	TAATACGACTCACTATAGGGAGGAAATCAAGGTTTGGCTGTA	429
		-R-	TAATACGACTCACTATAGGGTCATGCTGAGGTAAGCTTTCAA	
dsPpIMD	PPU01186-RA	-F-	TAATACGACTCACTATAGGGCTCCACGATCGGAAAATAGC	523
		-R-	TAATACGACTCACTATAGGGGTGCCACCTTTGTGCTTGTA	
dsLuc		-F-	TAATACGACTCACTATAGGGTGGAATGTTTACTACACTCG	
		-R-	TAATACGACTCACTATAGGGCATAATCATAGGACCTCTCA	
